# Study on Mixed Solvency Concept in Formulation Development of Aqueous Injection of Poorly Water Soluble Drug

**DOI:** 10.1155/2013/678132

**Published:** 2013-03-28

**Authors:** Shailendra Singh Solanki, Love Kumar Soni, Rajesh Kumar Maheshwari

**Affiliations:** ^1^School of Pharmacy, Devi Ahilya Vishwavidyalaya, UTD, Takshashila Campus, Indore 452001, India; ^2^Department of Pharmacy, SGSITS, Park Road, Indore 452003, India

## Abstract

In the present investigation, mixed-solvency approach has been applied for the enhancement of aqueous solubility of a poorly water- soluble drug, zaltoprofen (selected as a model drug), by making blends (keeping total concentrations 40% w/v, constant) of selected water-soluble substances from among the hydrotropes (urea, sodium benzoate, sodium citrate, nicotinamide); water-soluble solids (PEG-4000, PEG-6000); and co-solvents (propylene glycol, glycerine, PEG-200, PEG-400, PEG-600). Aqueous solubility of drug in case of selected blends (12 blends) ranged from 9.091 ± 0.011 mg/ml–43.055 ± 0.14 mg/ml (as compared to the solubility in distilled water 0.072 ± 0.012 mg/ml). The enhancement in the solubility of drug in a mixed solvent containing 10% sodium citrate, 5% sodium benzoate and 25 % S cosolvent (25% S cosolvent contains PEG200, PEG 400, PEG600, Glycerine and Propylene glycol) was more than 600 fold. This proved a synergistic enhancement in solubility of a poorly water-soluble drug due to mixed cosolvent effect. Each solubilized product was characterized by ultraviolet and infrared techniques. Various properties of solution such as pH, viscosity, specific gravity and surface tension were studied. The developed formulation was studied for physical and chemical stability. This mixed solvency shall prove definitely a boon for pharmaceutical industries for the development of dosage form of poorly water soluble drugs.

## 1. Introduction

Formulation has numerous benefits in drug discovery and development. It enables efficacy, toxicity, and pharmacokinetic (PK) studies. Formulation can improve oral bioavailability, shorten onset of a therapeutic effect, enhance stability of drugs, and reduce dosing frequency. More consistent dosing can be achieved by reducing food effect through formulation. Formulation can reduce side effects (i.e., decreasing tissue irritation and improving taste) [1–3]. An intramuscular (IM) medication is given by needle into the muscle. It can be a solution, oil, or suspension. Drugs in aqueous solution are rapidly absorbed. However, very slow constant absorption can be obtained if the drug is administered in oil or suspended in other repository vehicles. Solubility may also be enhanced by altering the pH and using cosolvents but excess amount of these agents may have adverse effects. The vehicle should contain a minimum amount and low concentration of the co-solvent to reduce viscosity and toxicity effects [3–6]. 

To significantly enhance the solubility of semipolar drugs, high concentrations of non-aqueous cosolvents may be required [7]. The formulation of solutions presents many technical problems to the industrial pharmacist. Special techniques are required to solubilize poorly water-soluble drugs. There are several techniques to enhance the aqueous solubilities of poorly water-soluble drugs; hydrotropy is one of such techniques. The term “hydrotropy” has been used to designate the increase in aqueous solubility of various poorly water-soluble compounds due to the presence of a large amount of additives. Concentrated aqueous hydrotropic solutions of urea, nicotinamide, sodium benzoate, sodium salicylate, sodium acetate, and sodium citrate have been observed to enhance the aqueous solubility of many poorly water-soluble drugs [8–12]. Melted (temperature less than 100°C) Polyethylene glycol (PEG) 4000, PEG 6000, and PEG 8000 dissolve diclofenac sodium (melting point: 283°C). This shows that melted PEGs act as solvents for diclofenac sodium. Melted urea (M.P.: 132–135°C) dissolves diclofenac sodium (M.P.: 283°C) [13]. This also shows that melted urea acts as solvent for diclofenac sodium. Melted ibuprofen (M.P.: 78°C) dissolves diclofenac sodium (M.P.: 283°C), salicylic acid (M.P.: 159°C), and niacinamide (M.P.: 132°C), which again shows that melted ibuprofen acts as solvent for diclofenac sodium, salicylic acid, and niacinamide, respectively. In supercritical fluid technology, liquefied carbon dioxide acts as solvent for many insoluble substances. These points indicate that all types of substances possess some solvent character [13, 14].

A drug administered in solution is immediately available for absorption and in most cases is more rapidly and efficiently absorbed than the same amount of drug administered in suspensions. Maheshwari et al. have demonstrated the synergistic solubilizing capability due to mixed-hydrotropy approach and this approach has been applied to analyze the poorly water-soluble drug, aceclofenac, titrimetrically. their research revealed application of hydrotropy in titrimetric and spectrophotometric estimations of a large number of poorly water-soluble drugs precluding the use of organic solvents [15–17]. They are of the opinion that hydrotropy is another type of co-solvency. Instead of using one solubilizer in a large concentration for a desired level of solubility, the concentrated solutions made by employment of several solubilizers in small concentrations may show additive or synergistic enhancement in solubility [18–22].

This mixed solvency concept may be utilized to prepare the concentrated (say 40% w/v or so, in strength) combined aqueous solutions of various water-soluble additives from the categories of the so-called hydrotropes (sodium benzoate, sodium ascorbate, sodium citrate, niacinamide, and urea), co-solvents (glycerin, propylene glycol, ethanol, PEG 200, 300, 400, and 600), water soluble solids (PVP, PEG 4000, 6000, 8000, and 10000), and cyclodextrins (beta-cyclodextrin and HP beta cyclodextrin) employing them in small, safe concentrations to solubilize the poorly water-soluble drugs to develop their dosage forms (solutions, syrups, injections, topical solutions, etc.). Therefore, the authors have proposed a mixed solvency approach for poorly water-soluble drugs. In the present investigation, the poorly water-soluble drug zaltoprofen has been selected as a model drug for formulating its aqueous injection by using mixed solvency approach [23–26]. 

Zaltoprofen obtained as white, crystals, or crystalline powder is freely soluble in acetone, soluble in methanol and ethanol (99.5), and practically insoluble in water (0.072 mg/mL), which precludes its use in parenteral formulations. It is gradually decomposed by light [27].

**Figure 1:**
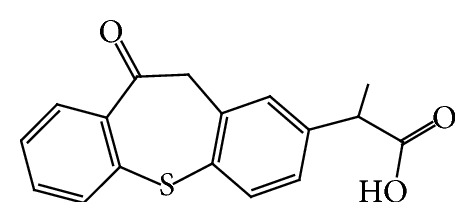
Structure of zaltoprofen.

Employing the concept of mixed solvency, attempts can be made to make a safe and strong solvent by proper choice of nontoxic solubilizers and, thus, the use of toxic organic solvents can be discouraged to a great extent (Figure 1).

## 2. Materials and Methods

### 2.1. Materials

Zaltoprofen was obtained as gift sample from Ipca Laboratories, Ratlam, India. Nicotinamide and propylene glycol were purchased from Loba Chemie, Mumbai, India. Sodium benzoate, urea, sodium citrate, glycerin, PEG 200, PEG 400, PEG 600, and PEG 6000 were obtained from Merck Chemicals Limited, Mumbai, India. All other chemicals and solvents used were of analytical/HPLC grade. Membrane filter (0.22 *μ*m) (Sartorius, Germany), aluminium seal, glass vials (5 mL), and rubber plugs (Modern Laboratories Indore, India) were also employed in this study. 

### 2.2. Estimation of Zaltoprofen

In the present investigation, UV spectrophotometric method was used for the estimation of zaltoprofen. The calibration curve of zaltoprofen was prepared in distilled water and various concentrations of water soluble solubilizers (hydrotropic agents and co-solvents) at 340 nm using double-beam spectrophotometer (UV-1800, Shimadzu, Japan) [12, 18, 27].

### 2.3. Solubility Determination

Solubility of zaltoprofen in various solutions was determined by equilibrium solubility method. Sufficient excess amount of zaltoprofen was added to 5 mL screw-capped glass vials containing buffers of pH (1.2–10.0), aqueous solution of hydrotropic agents, and different concentration of solubilizers (Tables 1 and 2). The vials were shaken mechanically for 12 h on mechanical shaker (Lab Hosp, Mumbai, India) at 37 ± 2°C. The solutions were allowed to equilibrate for the next 24 h. The solution was transferred into Eppendorf tubes and centrifuged for 5 min at 2000 rpm. The supernatants of each vial were filtered through 0.45 *μ* membrane filter (Pall Corporation, USA) and analyzed for drug content by UV visible spectrophotometer (UV-1800, Shimadzu, Japan) at 340 nm after appropriate dilutions. The study was performed in triplicate [24–26].

### 2.4. pH Dependent Solubility and Stability Determination

All the samples of saturated solution of drug at different pH were kept for 30 days at room temperature and analyzed for the drug content at 1, 7, 14, and 30th day to obtain the percentage of degradation and thereby to find the pH of maximum stability of zaltoprofen [24–26].

### 2.5. Method of Determination for Additive/Synergistic Effect on Solubility in Blends

An equilibrium solubility method was used to determine the additive or synergistic effect on solubility. The total strength of all solubilizers was 40% w/v (constant) in all aqueous mixed solvent systems (Table 3). The solubility of zaltoprofen was determined in these systems [26].

### 2.6. Properties of Mixed Solvent Solutions

Various properties of the solution such as pH, viscosity, specific gravity, and surface tension were studied using digital pH meter and Brookfield viscometer (Table 4) [23–25].

### 2.7. Drug Excipient Compatibility Studies

#### 2.7.1. UV Spectral Studies

To interpret the probable mechanism of solubilization, UV spectral studies of zaltoprofen were performed in different mixed solvent solutions to study the possible spectroscopic changes in the structure of zaltoprofen in the presence of different hydrotropes and co-solvents (Figures 2, 3, 4, 5, 6, 7, 8, 9, 10, and 11) [23–25].

#### 2.7.2. Fourier Transform Infrared (FTIR) Spectral Studies

FTIR spectra were obtained by means of an FTIR spectrophotometer (FTIR-8300S, Shimadzu, Japan). The samples were prepared by mixing of drug and potassium bromide in 1 : 1 ratio and measurements were attempted over the range of 400–4000 cm-1 (Figures 12 and 13 and Table 5) [23].

## 3. Formulation of Aqueous Injection

### 3.1. Preparation of Aseptic Area

The walls and floor of aseptic room were thoroughly washed with water and then disinfected by mopping with 2.5% v/v Dettol (Reckitt Benckiser Ltd., Kolkata, India) solution [23–25]. The bench was cleaned with 70% v/v isopropyl alcohol as well as sprayed in the atmosphere. The aseptic room was fumigated using a mixture of formaldehyde and potassium permanganate and the UV lights were switched on for 30 min prior to formulation of injections and the filling of injections into vials [28].

### 3.2. Treatment of Packing Material

Amber color glass vials of 2 mL capacity were washed several times with water then finally rinsed with distilled water. All these vials were placed in an inverted position and sterilized by dry heat in an oven at 180°C for 2 h. Rubber stoppers used for plugging the vials were first shaken in 0.2% liquid detergent solution for 2 h then washed several times with water to remove any detergent residue and finally rinsed with distilled water. These stoppers were sterilized by autoclaving at 15 lbs pressure (121°C temperature) for 20 min. Finally, the stoppers were rinsed with freshly prepared sterile distilled water and dried in vacuum oven under aseptic condition [23–26, 28].

### 3.3. Preparation of Aqueous Injection

On the basis of solubility data obtained from the final blends of mixed solvent, formulation of aqueous injection of zaltoprofen was prepared using mixed solvent system “F8.” This formulation contained 40 mg/mL zaltoprofen in mixed solvent system “F8” (10% sodium citrate, 5% sodium benzoate, and 25% w/v solvent system “S”). A 0.1% w/v sodium bisulfite was added as an antioxidant. Other additives like chelating agent and buffering agent were not included in these formulations as they might lead to change in the solubility behavior and upset the basic solubility enhancement ratio [23–26].

For the preparation of aqueous injection of zaltoprofen, 45 mL of solvent system “F8” solution was placed in 50 mL volumetric flask in the aseptic area and weighed amount of zaltoprofen and 0.1% w/v sodium metabisulfite were added as an antioxidant to the flask and were shaken for 1 h to ensure the complete dissolution of the former. pH of the solution was adjusted to 6−7.5 with 0.1 N HCl and 0.1 N NaOH solution, then the volume was made up to 50 mL with water for injection. These solutions were filtered through 0.45 *μ*m membrane filter (Pall Corporation, USA). The solutions were analyzed spectrophotometrically at 340 nm for drug content after appropriate dilutions with distilled water using the same vehicle as blank after appropriate dilution [23–25].

### 3.4. Aseptic Filtration and Packaging

The aqueous injection of zaltoprofen was sterilized by filtration through 0.2 *μ*m disposable membrane filter fitted in a holder of 5 mL glass syringe and the pressure on the piston was adjusted. After filtration, the preparation was packed by the sterilized air tight rubber closure and labeled. The final packed vials were sterilized by autoclaving at 15 lbs/sq. inch (121°C) for 15 min [24, 25].

## 4. Characterization of Aqueous Injection

### 4.1. Stability Study 

#### 4.1.1. Physical Stability Studies

The sealed or packed vials of the aqueous injections were visually inspected every day for 30 days against black and white backgrounds to see the changes occurring, if any, in physical appearance of aqueous injection like color, turbidity, pH, and so forth (Table 6), on storage at 2–8°C in a refrigerator, room temperature, and 40°C/75% RH in thermostatically controlled ovens (Lab Hosp, Mumbai, India).

#### 4.1.2. Chemical Stability Studies

The injection formulations were subjected to exhaustive chemical stability at 4 ± 2°C in a refrigerator and 37 ± 2°C and 60 ± 2°C in thermostatically controlled ovens for a period of 30 days. The formulations were analyzed spectrophotometrically initially and at particular intervals to calculate the drug content. The percent residual drug for each injection formulation at different time intervals as well as at different temperatures was calculated considering the initial drug content for each formulation to be 100% (Table 7). From the chemical stability data, the *K* values at 37 ± 2°C were determined. The time period required for 10% degradation of drug (*t*_10%_) or shelf life of formulation was calculated (Table 8) [23–25, 29].

## 5. Results and Discussion

As mentioned previously, the solubility of zaltoprofen in water is only 0.072 mg/mL, which is well below the target solution concentration of 1 mg/mL. The solubilizers selected for the present study possess a hydrophobic centre which can interact due to a large surface area and a mobile electron cloud known as a sextet. Thus these sites are available for non-bonded and the van der Waals interaction with water and drug. The molecules of water join to form cluster together. For solubilization the ionized solubilizers break this association and use the ion dipoles of water for salvation. Results showed that zaltoprofen was more soluble at alkaline pH than acidic pH when solubility studies of zaltoprofen at different pH were performed. This may be due to the acidic nature of zaltoprofen by virtue of its propionic acid group. Enhancement in solubility was found several-fold at pH 10.0 (Table 1) [30].

The solubility of zaltoprofen in different hydrotropic agents and co-solvents is showed in Table 2. The maximum solubility was observed in 2 M sodium benzoate with enhancement ratio of 306.30 and in 2 M nicotinamide with enhancement ratio of 227.43. Solubility enhancement for co-solvents could be ranked in decreasing order as PEG 600 > PEG 6000 > PEG 400 > PEG 200 > glycerin > propylene glycol. The solubility enhancement ratio for 2 M hydrotrope concentration was found to be 306.30 > 227.43 > 25.91 > 9.79, respectively (Table 2). 

The use of hydrotrope combinations yields higher zaltoprofen solubility than that of the single hydrotrope, and the higher the hydrotrope concentration in the solution, the more the drug that can be solubilized. The results show that the incorporation of hydrotropes in combination into cosolvent solution yields substantially greater drug solubility than that of cosolvent solution alone.

When we increase the concentration of solubilizers, there is increased insolubility of zaltoprofen but this increased level of solubilizer can have toxic effect. Therefore, the author further suggests that instead of taking a single solubilizer in large concentration (which may prove toxic) for development of a dosage form, a number of solubilizers may be taken in small concentrations (mixed solvency) curtailing their toxic level [24].

Results of mixed solvents system to determine saturated solubility showed that there is tremendous additive/synergistic effect on solubility which was 200–600-fold. The combination of 10% sodium citrate, 5% sodium benzoate, and 25% S cosolvent enhanced the solubility up to 600-fold. In fact, the drug solubility is well above the desired concentration. The solubility of drug in multiple cosolvent and hydrotrope mixtures is shown in Table 3. Saturated solution of drug was studied for pH dependent solubility and stability at room temperature and was analyzed for drug content, and results showed that the drug was stable at all pH ranges and drug content was found to be more than 98% after 30 days.

The various solution properties of mixed solvent F7–F10 were studied because these formulation blends showed solubility enhancement ratio up to 400–600-fold, that other blends were not selected due to less solubility enhancement ratio. pH of the formulations is less alkaline which could be less irritant to site of application. The viscosity of all the solutions was increased with increasing the slight changes in the concentration, while the surface tension and specific gravity of solutions were again slight deviated because of the change in concentration of solubilizers in solutions, and the results are shown in Table 4 [23].

**Figure 2:**
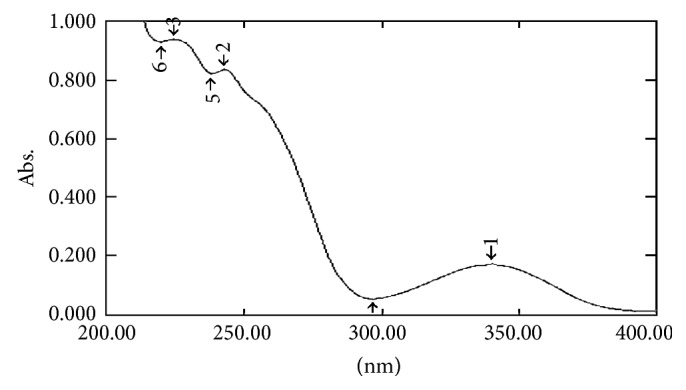
UV spectra of zaltoprofen in distilled water.

**Figure 3:**
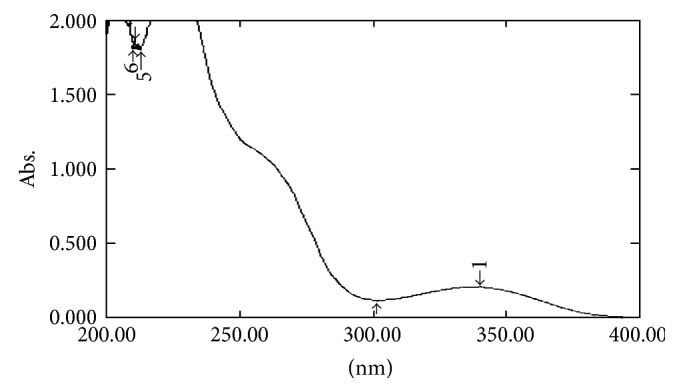
UV spectra of zaltoprofen in glycerin.

**Figure 4:**
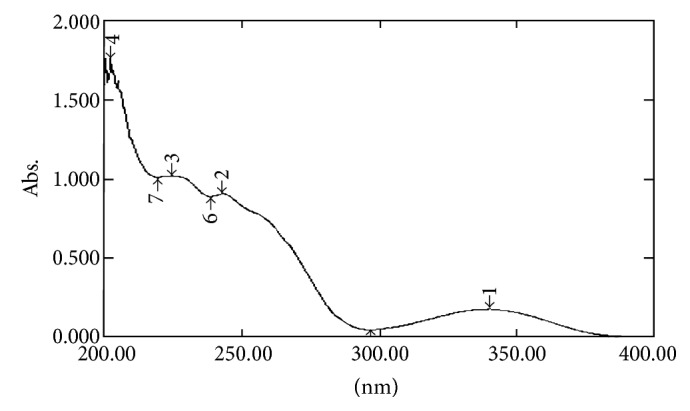
UV spectra of zaltoprofen in nicotinamide.

**Figure 5:**
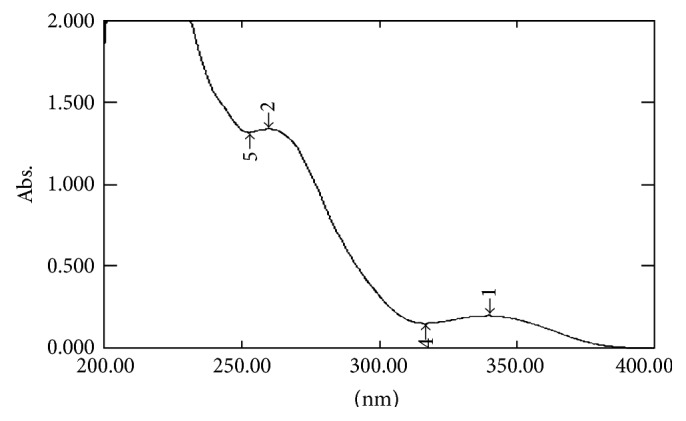
UV spectra of zaltoprofen in PEG 200.

**Figure 6:**
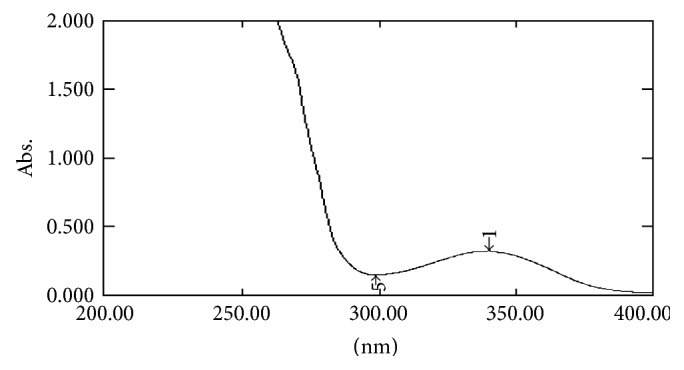
UV spectra of zaltoprofen in PEG 400.

**Figure 7:**
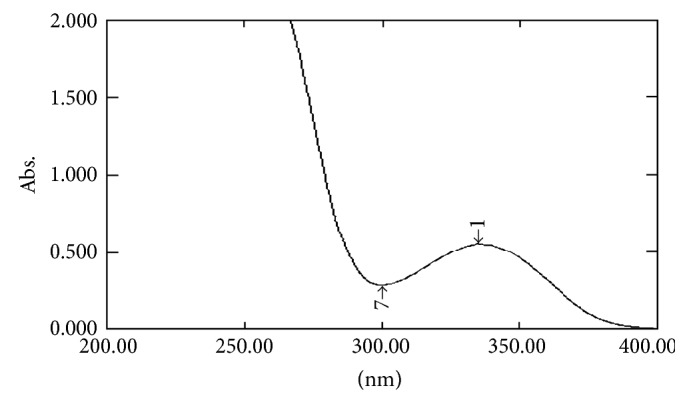
UV spectra of zaltoprofen in PEG 600.

**Figure 8:**
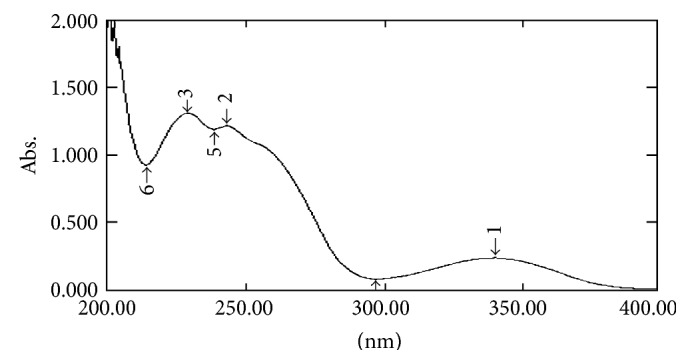
UV spectra of zaltoprofen in Propylene glycol.

**Figure 9:**
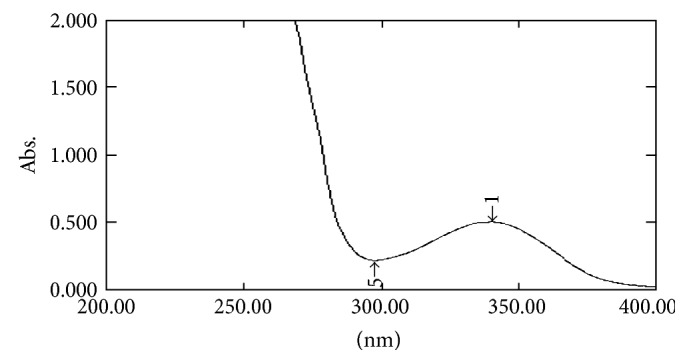
UV spectra of zaltoprofen in sodium citrate.

**Figure 10:**
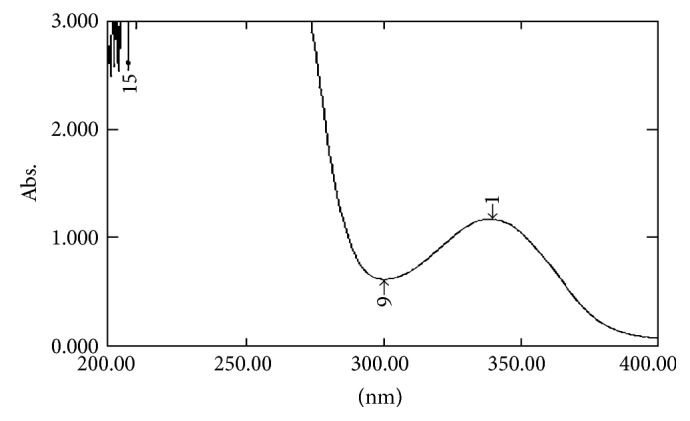
UV spectra of zaltoprofen in urea.

**Figure 11:**
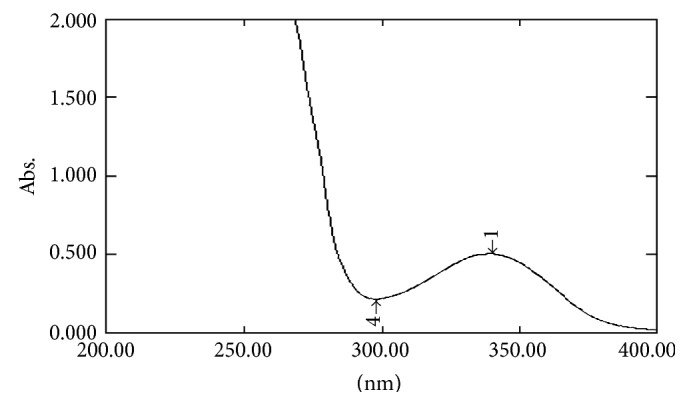
UV spectra of zaltoprofen in sodium benzoate.

**Figure 12:**
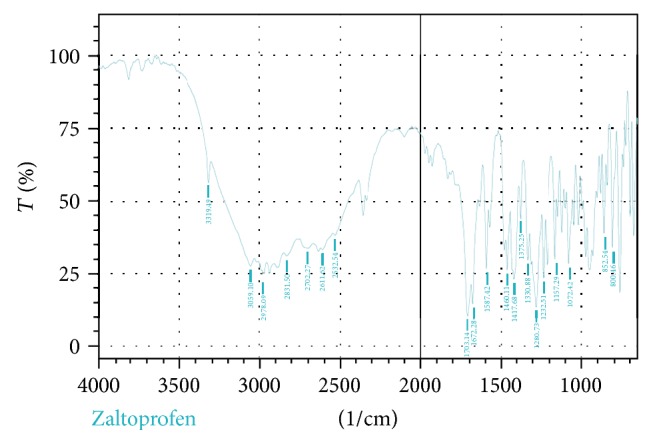
FTIR spectrum of zaltoprofen.

**Figure 13:**
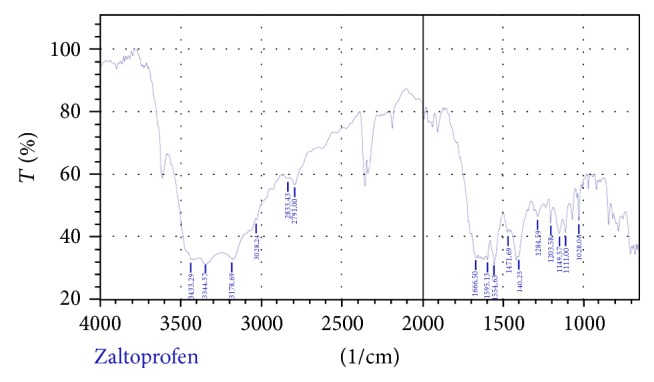
FTIR spectrum of zaltoprofen with all the solubilizers.

The UV absorption spectra of zaltoprofen in various solubilizers solutions showed a slight shift in *λ*_max_ (340 ± 1 nm), which can be due to minor electronic changes in the structure of drug molecules. Small additional peaks of solubilizers and cosolvent were also observed in the figure of UV spectra which indicates that there is no interference of *λ*_max_ of solubilizer/cosolvents with the *λ*_max_ of the drug (Figures 2–11). In order to check the integrity of the drug in the formulation, an IR spectral study was carried out. The spectra obtained from IR studies at wavelength from 4000 cm^−1^ to 400 cm^−1^ showed that there are neither major shifts nor any loss of functional peaks between the spectra of the drug and polymer. IR of the drug with all solubilizer was compared with that of the pure drug to see whether there was any change in the structure or interaction with the polymers, and results of FTIR spectral analysis showed no evidence of strong complex formation and integrity of the drug is not affected (Figures 12 and 13 and Table 5) [23].

Blend F8 was selected and used to develop aqueous parenteral formulation due to its superior and synergistic effect on solubility of zaltoprofen. Formulated aqueous injection was subjected to physical stability testing at 2–8°C, at room temperature, and at 40°C/75% RH. Results of the physical stability study of formulation F8 showed that it remains unchanged with respect to pH, color stability, and no turbidity or precipitate formation was observed at different storage conditions. Prepared formulation has shown appreciable physical stability (Table 6). The data on chemical stability at different temperatures and time intervals are shown in Table 7 which showed that the degradation of zaltoprofen follows first-order kinetics. The calculated *K* values, that is, decomposition rate constant of formulations, are reported in Table 8. The time required for the 10% degradation of drug for formulation was calculated. The results show that the prepared formulations had a shelf life of 253.31 days.

## 6. Conclusion

In conclusion, the results of this study suggest that a stable aqueous injection of zaltoprofen and other poorly water-soluble drug can be successfully developed using the concept of mixed-solvency approach. The amount of individual solubilizer required to increase the measurable solubility shall be very high which sometimes shows the toxicity. Therefore, the use of blends of solubilizers (sodium benzoate, nicotinamide, sodium citrate, urea, and cosolvents like PEG, glycerine, and propylene glycol) which are physiologically compatible often acts synergistically to improve the solubility and reduce the risk of toxicity.

The results of the present investigation showed the possibility of aqueous injection of poorly water-soluble drugs using combination of various solubilizers and hydrotropic agents which act synergistically at very low individual concentrations. Hence, toxicity and safety related issues may not raise concern and would suggest their adoptability for large-scale manufacturing. The proposed techniques would be economical, convenient, and safe. Thus, this study opens the chance of preparing aqueous formulations of poorly water-soluble drugs, if chemical stability of the drug remains unaffected. Mixed solvency approach produces a physical stable formulation which results in the administration of low level of cosolvents to the patient, thus reducing or eliminating the effect of cosolvent toxicity and erythrocyte damage. 

Thus it can be concluded that, with the carefully designed experimental technique, solubility of poorly water-soluble drug can be improved by using the “mixed solvency” approach. The application of the mixed solvency approach in the development of formulations shall prove to be a boon for pharmaceutical industries because the quantities of water soluble solubilizers present in the blends can be selected at safe level (well below their toxic levels) for a modest increase in solubility of a water-insoluble drug.

## Figures and Tables

**Table 1:** pH dependent solubility of zaltoprofen at room temperature.

Buffer solutions	Solubility∗ (mg/mL)
Distilled water	0.072 ± 0.012
Hydrochloric acid buffer pH 1.2	0.0134 ± 0.018
Hydrochloric acid buffer pH 2.2	0.0196 ± 0.006
Acid phthalate buffer pH 4.0	0.0285 ± 0.014
Phosphate buffer pH 5.8	0.0384 ± 0.007
Phosphate buffer pH 8.0	0.2067 ± 0.013
Alkaline borate buffer pH 9.0	1.0241 ± 0.12
Alkaline borate buffer pH 10.0	1.376 ± 0.011

^*^Average of 3 determinations.

**Table 2:** Solubility enhancement ratio of zaltoprofen in different solubilizers at 37 ± 2°C.

Solvents	Solubility∗ (mg/mL)	Enhancement ratio
Distilled water	0.072 ± 0.012	—
2 M sodium benzoate	22.054 ± 0.008	306.30
2 M urea	1.866 ± 0.012	25.91
2 M nicotinamide	16.375 ± 0.14	227.43
2 M sodium citrate	0.705 ± 0.08	9.79
20% w/v propylene glycol	0.298 ± 0.14	4.13
40% w/v propylene glycol	0.653 ± 0.12	9.06
20% w/v glycerine	0.538 ± 0.18	7.47
40% w/v glycerine	1.022 ± 0.11	14.19
20% w/v PEG 6000	0.671 ± 0.12	9.31
40% w/v PEG 6000	1.343 ± 0.15	18.65
20% w/v PEG 200	0.496 ± 0.06	6.88
40% w/v PEG 200	1.025 ± 0.17	14.23
20% w/v PEG 400	0.624 ± 0.11	8.66
40% w/v PEG 400	1.293 ± 0.05	17.95
20% w/v PEG 600	0.782 ± 0.12	10.86
40% w/v PEG 600	1.358 ± 0.009	18.86

^*^Average of 3 determinations.

**Table 3:** Mixed solvent for saturated solubility determination of zaltoprofen.

Blend code	Solvents	Solubility∗ (mg/mL)	Enhancement ratio
A1	Distill water	0.072 ± 0.012	—
F1	15% SB + 25% S	16.80 ± 0.008	233.33
F2	15% UR + 25% S	9.091 ± 0.011	126.26
F3	15% N + 25% S	12.77 ± 0.013	177.36
F4	15% SC + 25% S	19.652 ± 0.009	272.94
F5	10% SB + 5% UR + 25% S	26.87 ± 0.007	373.19
F6	10% UR + 5% N + 25% S	10.625 ± 0.008	147.54
F7	10% N + 5% SC + 25% S	37.77 ± 0.12	524.58
F8	10% SC + 5% SB + 25% S	43.055 ± 0.14	597.98
F9	10% SB + 5% N + 25% S	34.583 ± 0.12	480.31
F10	10% SB + 5% SC + 25% S	28.33 ± 0.15	393.47
F11	10% N + 5% UR + 25% S	26.14 ± 0.021	363.05
F12	10% N + 5% SB + 25% S	27.08 ± 0.28	376.11

^*^Average of 3 determinations.

**Table 4:** Properties of the optimized blends.

Experiment blends	pH	Viscosity (cps)	Surface tension (dynes/cm)	Specific gravity
F7	7.14	6.21	51.21	1.053
F8	7.18	6.54	53.12	1.082
F9	7.11	6.42	52.38	1.071
F10	7.28	6.46	54.11	1.062

**Table 5:** Characteristic infrared absorptions of functional groups.

Range cm^−1^		Functional group
Drug	Drug with solubilizers
3019.98, 3000.69	3019.98, 3000.69	Aromatic C–H stretching
1800–2400	1800–2400	Aromatic overtone
1600–1650	1600–1650	Ketonic group
1580–1600	1580–1600	C–C multiple bond str. (aromatic)
1725–1700	1725–1700	Saturated acid (aliphatic)
600	600	C–S str.
2500–3200	2500–3200	–OH of acid

**Table 6:** Physical stability data of formulation F8.

	Physical stability parameter					
Condition	pH		Color		Precipitation	
	Initial	After 30 days	Initial	After 30 days	Initial	After 30 days
Refrigeration (2–8°C)	7.18	7.15	Colorless	Colorless	No ppt.	No ppt.
Room temperature	7.18	7.17	Colorless	Colorless	No ppt.	No ppt.
40°C/75% RH	7.18	7.21	Colorless	Colorless	No ppt.	No ppt.

**Table 7:** Chemical stability data of formulation F8.

Formulation code	Temperature	Concentration∗ mg/vials				Degradation
		0 day	7 day	15 day	30 day
	4 ± 2°C	80.04 ± 0.012	79.41 ± 0.013	79.03 ± 0.011	79.06 ± 0.014	1.18%
F8	37 ± 2°C	80.04 ± 0.012	79.22 ± 0.011	78.94 ± 0.014	78.87 ± 0.020	1.42%
	60 ± 2°C	80.04 ± 0.009	78.84 ± 0.021	78.47 ± 0.019	78.59 ± 0.012	1.77%

^*^Average of 3 determinations.

**Table 8:** Degradation rate constant and shelf life of formulated products.

Formulation	Degradation rate constant *K* (days^−1^) ×10^−4^ at 37 ± 2°C	Shelf life at 37 ± 2°C
Zaltoprofen injection	4.145 × 10^−4^	253.31 days

## References

[1] Neervannan S. (2006). Preclinical formulations for discovery and toxicology: physicochemical challenges. *Expert Opinion on Drug Metabolism and Toxicology*.

[2] Simamora P., Pinsuwan S., Alvarez J. M., Myrdal P. B., Yalkowsky S. H. (1995). Effect of pH on injection phlebitis. *Journal of Pharmaceutical Sciences*.

[3] Lee Y. C., Zocharski P. D., Samas B. (2003). An intravenous formulation decision tree for discovery compound formulation development. *International Journal of Pharmaceutics*.

[4] Sweetana S., Akers M. J. (1996). Solubility principles and practices for parenteral drug dosage form development. *PDA Journal of Pharmaceutical Science and Technology*.

[5] Strickley R. G. (2004). Solubilizing excipients in oral and injectable formulations. *Pharmaceutical Research*.

[6] Yalkowsky S. H., Roseman T. J., Yalkowsky S. H. (1981). Solubilization of drugs by co-solvents. *Techniques of Solubilization of Drugs*.

[7] Gould P. L., Goodman M., Hanson P. A. (1984). Investigation of the solubility relationships of polar, semi-polar and non-polar drugs in mixed co-solvent systems. *International Journal of Pharmaceutics*.

[8] Maheshwari R. K. (2005). Analysis of frusemide by application of hydrotropic solublization phenomenon. *Indian Pharmacist*.

[9] Maheshwari R. K. (2005). New application of hydrotropic solublization in the spectrophotometric estimation of ketoprofen in tablet dosage form. *Pharma Review*.

[10] Maheshwari R. K. (2006). A novel application of hydrotropic solubilization in the analysis of bulk samples of ketoprofen and salicylic acid. *Asian Journal of Chemistry*.

[11] Maheshwari R. K. (2006). Novel application of hydrotropic solubilization in the spectrophotometric analysis of tinidazole in dosage form. *Asian Journal of Chemistry*.

[12] Maheshwari R. K. (2006). Application of hydrotropic solubilization in the analysis of aceclofenac drug. *Asian Journal of Chemistry*.

[13] Maheshwari R. K. (2009). Solubilization of ibuprofen by mixed solvency approach. *The Indian Pharmacist*.

[14] Maheshwari R. K. (2010). Potentiation of solvent character by mixed solvency concept: a novel concept of solubilization. *Journal of Pharmceutical Research*.

[15] Maheshwari R. K., Chaturvedi S. C., Jain N. K. (2005). Application of hydrotropic solubilization phenomenon in spectrophotometric analysis of hydrochlorothiazide tablets. *Indian Drugs*.

[16] Maheshwari R. K., Chaturvedi S. C., Jain N. K. (2006). Titrimetric analysis of aceclofenac in tablets using hydrotropic solubilization technique. *Indian Drugs*.

[17] Maheshwari R. K., Bishnoi S. R., Kumar D. (2008). Spectrophotometric analysis of hydrochlorothiazide tablets using chlorpheniramine maleate as hydrotropic solubilizing agent. *Asian Journal of Chemistry*.

[18] Maheshwari R. K. (2006). Application of hydrotropic solubilization phenomenon in spectrophotometric estimation of norfloxacin in tablets. *Indian Journal of Pharmaceutical Education and Research*.

[19] Maheshwari R. K. (2006). Novel application of hydrotropic solubilization in the spectrophotometric analysis of piroxicam in solid dosage form. *Indian Drugs*.

[20] Aher K. B., Bhavar G. B., Joshi H. P., Chaudhari S. R. (2011). Economical spectrophotometric method for estimation of zaltoprofen in pharmaceutical formulations. *Pharmaceutical Methods*.

[21] Maheshwari R. K. (2005). Spectrophotometric determination of cefixime in tablets by hydrotropic solubilization phenomenon. *The Indian Pharmacist*.

[22] Maheshwari R. K., Singh M. (2008). Quantitative determination of ketoprofen bulk drug using sodium salt of aspirin as hydrotropic solubilizing agent. *Asian Journal of Chemistry*.

[23] Jain A. K. (2008). Solubilization of indomethacin using hydrotropes for aqueous injection. *European Journal of Pharmceutics and Biopharmaceutics*.

[24] Agrawal S., Pancholi S. S., Jain N. K., Agrawal G. P. (2004). Hydrotropic solubilization of nimesulide for parenteral administration. *International Journal of Pharmaceutics*.

[25] Maheshwari R. K., Shilpkar R. (2012). Formulation development and evaluation of injection of poorly soluble drug using mixed solvency concept. *International Journal of Pharmacy and Biological Science*.

[26] Maheshwari R. K. (2009). ‘Mixed-Solvency’-a novel concept for solubilization of poorly water- soluble drugs. *Delving Journal*.

[27] (2006). The minister of health labour and welfare. *Japanese Pharmacopoeia*.

[28] Dicosta J., Khan S. (2011). Calculation for aceclofenac injection using hydrotropic solubilization technique. *International Journal of Pharmaceutical Science and Health Care*.

[29] Martin A. (2006). *Solubility and Distribution Phenomena, Physical Pharmacy and Pharmaceutical Sciences*.

[30] Saleh A. M., Badwan A. A., El Khordagui L. K. (1983). A study of hydrotropic salts, cyclohexanol and water systems. *International Journal of Pharmaceutics*.

